# Insensitivity to Fearful Emotion for Early ERP Components in High Autistic Tendency Is Associated with Lower Magnocellular Efficiency

**DOI:** 10.3389/fnhum.2017.00495

**Published:** 2017-10-12

**Authors:** Adelaide Burt, Laila Hugrass, Tash Frith-Belvedere, David Crewther

**Affiliations:** Centre for Human Psychopharmacology, Faculty of Health, Arts and Design, Swinburne University of Technology, Melbourne, VIC, Australia

**Keywords:** spatial frequency, event related potentials, P100, autistic tendency, fearful face perception, magnocellular inefficiency

## Abstract

Low spatial frequency (LSF) visual information is extracted rapidly from fearful faces, suggesting magnocellular involvement. Autistic phenotypes demonstrate altered magnocellular processing, which we propose contributes to a decreased P100 evoked response to LSF fearful faces. Here, we investigated whether rapid processing of fearful facial expressions differs for groups of neurotypical adults with low and high scores on the Autistic Spectrum Quotient (AQ). We created hybrid face stimuli with low and high spatial frequency filtered, fearful, and neutral expressions. Fearful faces produced higher amplitude P100 responses than neutral faces in the low AQ group, particularly when the hybrid face contained a LSF fearful expression. By contrast, there was no effect of fearful expression on P100 amplitude in the high AQ group. Consistent with evidence linking magnocellular differences with autistic personality traits, our non-linear VEP results showed that the high AQ group had higher amplitude K2.1 responses than the low AQ group, which is indicative of less efficient magnocellular recovery. Our results suggest that magnocellular LSF processing of a human face may be the initial visual cue used to rapidly and automatically detect fear, but that this cue functions atypically in those with high autistic tendency.

## Introduction

Autism spectrum disorder (ASD) is a broad group of disorders, characterized by impairments in communication and social awareness, and by repetitive stereotyped behaviors (American Psychiatric Association, 2013). In addition to these well-known impairments, there are some differences in perceptual functioning, particularly in the visual domain (Dakin and Frith, 2005; Kellerman et al., 2005; Pellicano et al., 2005; Dale and Salt, 2008; McCleery et al., 2009; Simmons et al., 2009). These perceptual differences extend to the neurotypical population (Almeida et al., 2010; Sutherland and Crewther, 2010; Jackson et al., 2013) for individuals with high scores on the Autism Spectrum Quotient (AQ) personality scale (Baron-Cohen et al., 2001). This suggests that the underlying physiology of ASD may be distributed throughout a broader autistic phenotype, which contributes to visual differences in these individuals (Bailey et al., 1995; Braddick et al., 2003).

Detection of potentially threatening stimuli, such as fearful or angry faces involves a distributed network of cortical and subcortical regions (LeDoux, 1998; Johnson, 2005; Kragel et al., 2016). Several researchers have proposed that rapid processing of threat occurs via a direct, subcortical route from the pulvinar and superior colliculus to the amygdala (LeDoux, 1998; Morris et al., 1998a; Öhman, 2005). Direct evidence of a subcortical (retino-collicular-pulvinar) route to the amygdala has come from animal studies (LeDoux, 1998), whereas functional evidence in humans has been inferred based on evidence of emotional face processing in cortically blind participants (Morris et al., 2001), unconscious processing of emotional stimuli (Morris et al., 1998a), and magnetoencephalographic (MEG) responses to fearful stimuli in the amygdala and subcortical structures (Streit et al., 2003). Clear anatomical evidence from *in vivo* DTI tractography conducted in humans demonstrates connections between the amygdala and superior colliculus (Rafal et al., 2015); and amygdala and pulvinar (Tamietto et al., 2012). In support of this model, connectivity analyses have shown that fearful stimuli engage a distributed network of brain sites including the bilateral fusiform gyrus, dorsal/anterior precuneus, amygdala, hippocampus, and parahippocampal regions (Kragel and LaBar, 2015).

In order to compare cortical and subcortical routes for affective information processing, several researchers have taken advantage of the tuning properties of cells in the subcortical structures (Vuilleumier et al., 2003; Öhman, 2005; Vlamings et al., 2009). Cells in the input layers of the superior colliculus receive input from the magnocellular pathway (Schiller et al., 1979). Magnocellular neurons have relatively large receptive fields and respond preferentially to low spatial frequency (LSF) input, compared with parvocellular neurons which have smaller receptive fields and respond preferentially to high spatial frequency (HSF) input (Livingstone and Hubel, 1988). Processing of coarse information contained in the LPFs precedes processing of more detailed information contained in the HSFs, with magnocellular input reaching V1 25–31 ms earlier than parvocellular input (Klistorner et al., 1997; Bullier, 2001; Bar et al., 2006; Sutherland and Crewther, 2010; Crewther et al., 2016). This temporal precedence has been termed the ‘magnocellular advantage’ (Laycock et al., 2007).

In support of a magnocellular route for fearful face processing, Vuilleumier et al. (2003) found enhanced BOLD responses in the superior colliculus and amygdala structures for LSF, but not HSF, fearful expressions. However, the amygdala also receives input from the anterior inferotemporal cortex (Aggleton, 1993), so it is not ‘blind’ to HSF fearful face representations. In fact, the amygdala is likely to have numerous connections with subcortical and cortical structures, which process and integrate both LSF and HSF, to form a ‘whole’ fearful face percept. Evidence for multiple pathways comes from connectivity analyses demonstrating that fearful stimuli engage a distributed network of brain sites including the bilateral fusiform gyrus, dorsal/anterior precuneus, amygdala, hippocampus, and parahippocampal regions (Kragel and LaBar, 2015). In addition, evidence for multiple pathways is presented by one fMRI study demonstrating that during unconscious processing using binocular rivalry and backward masking, when visual input must be processed via a subcortical route, the amygdala has limited capacity to differentiate between facial emotions (Morris et al., 1998b; Williams et al., 2004).

Sets of hybrid face stimuli have been used in numerous studies in order to investigate the relative contributions of LSF and HSF information to various aspects of emotional face processing, while controlling the natural variation of spatial frequencies within the images (Pourtois et al., 2005; Laeng et al., 2010, 2013; Prete et al., 2014, 2015a,b,c, 2016). LSF information generally includes coarse, global visual features whereas HSF information includes finer, more detailed features (De Valois and De Valois, 1988). Hybrid face stimuli are created from different combinations of the LSF and HSF filtered components of neutral and emotional faces. By equating the luminance and contrast of LSF and HSF within one image, only the change in emotional expression of the face remains (Pourtois et al., 2005). Hybrid faces have been used to assess conscious report of fearful face perception, where HSF fearful and neutral expressions are more rapidly discriminated than LSF expressions (Stein et al., 2014). However, behavioral report does not necessarily reflect the rapid, neural processing that occurs prior to conscious perception of a fearful face, which can be investigated with electroencephalogram (EEG). For consistency, throughout this paper we will refer to hybrids created from LSF and HSF, fearful (F) and neutral (N) face stimuli as follows: F_LSF_–F_HSF_, F_LSF_–N_HSF_, N_LSF_–F_HSF_, and N_LSF_–N_HSF_.

To elucidate the time course of rapid fear perception, electrophysiological studies have primarily focused on the P100 and N170 waveforms. The visual P100 is a fast response, typically evoked between 90 and 140 ms, that is maximal over lateral–occipital–parietal sites, and appears to originate from striate and extrastriate neural generators (Clark and Hillyard, 1996; Mangun et al., 1997; Allison et al., 1999). Cueing studies suggest the P100 amplitude is an index of the attentional gain mechanisms that suppress responses to irrelevant stimuli (Hillyard and Anllo-Vento, 1998). In addition, the P100 is sensitive to affect, with greater amplitude responses to fearful face presentation, compared to other emotions including neutral, happy, angry, sad, disgust, and surprise faces (Pizzagalli et al., 1999; Batty and Taylor, 2003; Pourtois et al., 2004; Magnée et al., 2008; Feng et al., 2009; Forscher and Li, 2012; Meaux et al., 2013; Smith et al., 2013; Zhang et al., 2013). The N170 is negative peak that occurs approximately 170 ms post-stimulus, originating from a network of face/object processing regions including the fusiform gyrus, superior temporal sulcus and inferior, middle and superior temporal gyri (Henson et al., 2003). N170 amplitude is sensitive to configural processing of faces, as evidenced by higher amplitude response to upright over inverted faces (Bentin et al., 1996). Affective input also modulates these early waveforms, with greater amplitude P100 and N170 responses to fearful or angry expressions (Batty and Taylor, 2003; Pegna et al., 2008).

Several studies have investigated whether fearful face modulation of visual ERPs relies on LSF (i.e., magnocellular) input. The P100 amplitude is enhanced for F_LSF_–N_HSF_ hybrid faces compared to N_LSF_–N_HSF_ hybrid faces in the right hemisphere, but not for N_LSF_–F_HSF_ hybrids (Pourtois et al., 2005); however, this effect is only observed when the images have been equated for luminance and contrast (Vlamings et al., 2009). In addition, Vlamings et al. (2009) found shorter P100 latency for F_LSF_–N_HSF_ compared to N_LSF_–F_HSF_ hybrids, which trended toward significance in the fearful expression condition. Findings regarding the effects of emotion and spatial frequency on the N170 amplitude have been mixed. Some studies reported no effect of emotion on the N170 response (Holmes et al., 2005; Pourtois et al., 2005), however, Vlamings et al. (2009) reported N170 enhancement for F_LSF_–N_HSF_ but not N_LSF_–F_HSF_ faces, regardless of whether the stimuli were equated for luminance and contrast.

Early electrophysiological responses to fearful faces vary across the autistic spectrum, indicating there are individual differences in the neural pathways that produce these responses. In autistic individuals, the P100 tends to be delayed, reduced in amplitude and less lateralized than in neurotypical groups (Bailey et al., 2005; Wong et al., 2008; McCleery et al., 2009; Luo et al., 2010; Batty et al., 2011; Fujita et al., 2013; Tye et al., 2013; Wagner et al., 2013; Key and Corbett, 2014; Bonnard-Couton et al., 2015; Lassalle and Itier, 2015; Anzures et al., 2016). In an investigation of face processing in the broader autistic phenotype, Stavropoulos et al. (2016) found that P100 and N170 latencies tended to be slower and decreased for people with high AQ scores, compared to people with low AQ scores. They found that non-consciously perceived emotional faces elicited enhanced neural responses regardless of AQ score, yet they did not use hybrid faces so it is unclear whether the two groups utilized LSF information in the same way. De Jong et al. (2008) investigated the effects of gaze cueing on ERPs and found that for neurotypical observers, gaze cueing had a stronger effect on ERPs for LSF filtered faces; whereas for autistic observers, cueing effects were stronger for HSF filtered faces. These findings indicate that the P100 and N170 responses to fearful emotional faces may be particularly affected in individuals with high autistic tendency. Therefore, we aimed to compare the effects of LSF and HSF fearful expressions on ERP responses for high and low AQ groups.

Several visual studies provide evidence for magnocellular/dorsal stream abnormalities in ASD (Pellicano et al., 2005; Milne et al., 2006; McCleery et al., 2009) and in the broader autistic phenotype (Almeida et al., 2010; Sutherland and Crewther, 2010; Jackson et al., 2013; Thompson et al., 2015; Stavropoulos et al., 2016). Other studies have indicated that the relationship between magnocellular function and ASD is more complicated (Bertone et al., 2003; Del Viva et al., 2006). The current view is that ASD involves complex interactions between multiple visual pathways, rather than a specific magnocellular/dorsal stream impairment (Simmons et al., 2009; Thye et al., 2017). Despite the nature of magnocellular impairment remaining unresolved, there are differences in the rapid output of magnocellular neurons to V1 in high autistic tendency (Sutherland and Crewther, 2010; Jackson et al., 2013). Moreover, as discussed above, magnocellular neurons have a preference for LSF input, resulting in rapid V1 activation. Neural efficiency of magnocellular neurons can be studied through Weiner kernel analysis of multifocal visual evoked potentials (mfVEP) (Sutherland and Crewther, 2010; Jackson et al., 2013). The first slice of the second order, non-linear VEP kernel (K2.1) measures non-linearity in neural recovery (referenced one video frame back). Studies of the contrast response function indicate that the N60 K2.1 waveform, and early components (with the same latency) of the K2.2 waveform (second slice- referenced two frames back) are of magnocellular origin (Baseler and Sutter, 1997). Higher amplitudes of these waveforms indicate a greater degree of inefficiency in neural recovery by magnocellular neurons. The amplitude of the N60 negativity in the first slice of the second order kernel (K2.1) is elevated in high AQ observers, indicative of poor magnocellular recovery rate (Jackson et al., 2013). Hence, Jackson et al.’s (2013) findings are consistent with the lack of efficiency of the magnocellular pathway in ASD.

Based on prior research by Vlamings et al. (2009) we hypothesized that the low AQ group would exhibit significantly higher amplitude and shorter latency P100 and N170 responses to F_HSF_–F_LSF_ hybrids compared to N_HSF_–N_LSF_ hybrids, and to F_LSF_–N_HSF_ compared to F_HSF_–N_LSF_ hybrids. Based on the findings by Stavropoulos et al. (2016), it was hypothesized that the P100 and N170 would be lower for the high AQ group than for the low AQ group. Furthermore, we predicted that F_LSF_–N_HSF_ expressions would have a greater influence on response amplitudes in the low AQ group than in the high AQ group. We aimed to use non-linear VEP to assess magnocellular recovery, as indexed by the amplitude of the K2.1 waveform. It was hypothesized, the K2.1 response amplitude would be higher in the high AQ group, which is indicative of inefficient recovery rate of neurons within the magnocellular system.

## Materials and Methods

### Participants

Participants were recruited through advertising and social media to complete an online AQ survey (Baron-Cohen et al., 2001). Thirty-seven participants with normal or corrected to normal vision completed the hybrid face EEG study conducted at Swinburne University of Technology, Melbourne, Australia. Two participants were excluded from the analysis because of very high movement artifact (one recording was actually discontinued for this reason), and a further two participants were excluded because their scores were in the mid-range of AQ (which we only established after recording). The final sample included 16 low AQ participants (3 male; 13 female; *M* = 24.19 years, *SD* = 5.41) and 17 high AQ participants (11 male; 6 female; *M* = 25.29 years, *SD* = 5.72). MfVEP data collection commenced after we had already began collecting data for the facial emotion study; hence only 12 low AQ and 12 high AQ participants completed the mfVEP recordings. For the behavioral data, two participants were excluded due to corrupted data files and a further participant was excluded due to misunderstanding task instructions, leaving a sample of 31 participants (17 high AQ and 14 low AQ). All participants gave informed consent in accordance with the Declaration of Helsinki, and the Swinburne Human Research Ethics Committee approved the study.

### Autistic Quotient Online Survey

The AQ (Baron-Cohen et al., 2001) is a 50-item measure of autistic trait levels across the normal adult population. Low and high group cut-offs for the EEG study were based on the population mean (*M* = 17, *SD* = 6) for the AQ groups (Ruzich et al., 2015). The low AQ group (*n* = 16) had a mean AQ score of 6.88 (*SD* = 3.22) and the high AQ (*n* = 17) group had a mean AQ score of 29.00 (*SD* = 5.95).

### Hybrid Face Stimuli

Fourteen images of neutral and fearful faces were selected from the NimStim Face Set (Tottenham et al., 2009). The images were transformed to gray-scale and cropped with a smoothed edge to remove external features (e.g., hair, neck) using Adobe Photoshop. The fearful faces were altered to have a 30% increase in pupil size, to reproduce a physiologically accurate fearful response (Demos et al., 2008).

**FIGURE 1:**
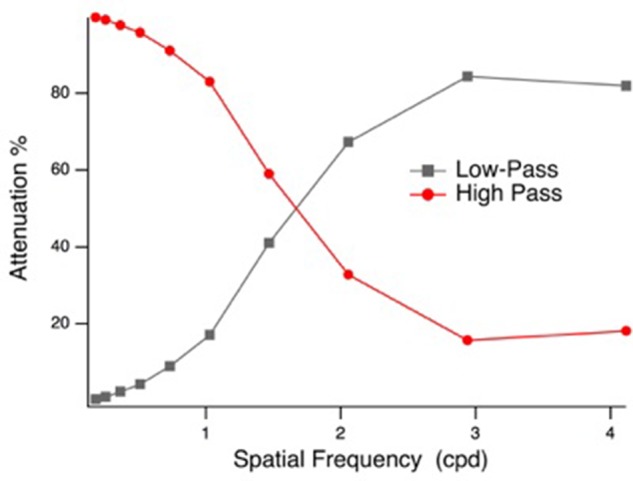
High-pass and low-pass filters: spatial frequency (cpd) vs. attenuation (%) of signal.

**FIGURE 2:**
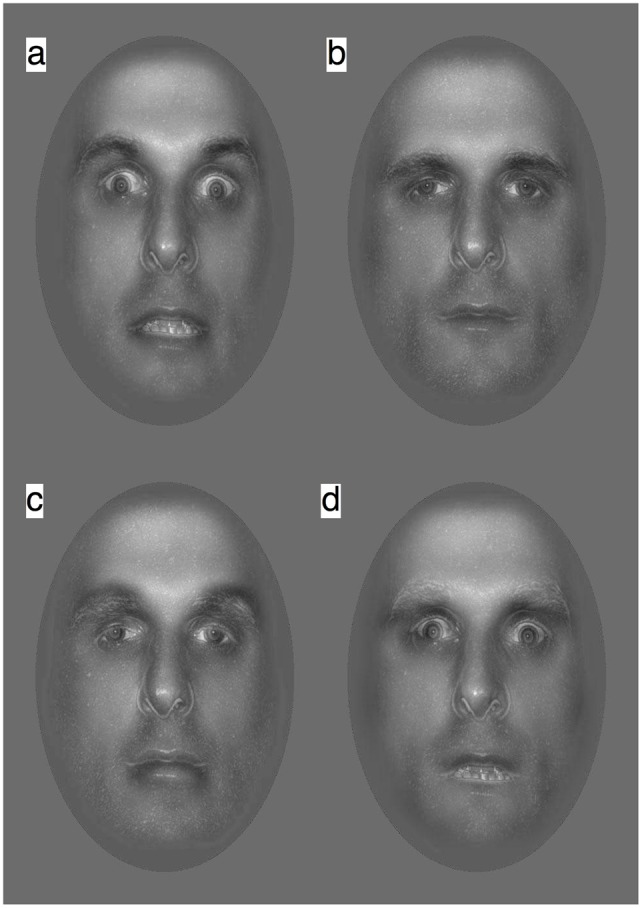
Hybrid face stimuli were created by recombining the LSF and HSF components of fearful (F) and neutral (N) faces into hybrids as: **(a)** F_LSF_–F_HSF_ **(b)** N_LSF_–N_HSF_ **(c)** F_LSF_–N_HSF_, and **(d)** N_LSF_–F_HSF_.

Face stimuli were spatial frequency filtered (Gaussian blur, low-pass filter preserving spatial frequencies <2 cpd and high pass filter preserving spatial frequencies >1.9 cpd, based on the point of 1 octave attenuation) using Photoshop (Adobe Systems Inc., San Jose, CA, United States), similar to prior investigations (Schyns and Oliva, 1999; Vlamings et al., 2009). The high- and low-pass filter characteristics are illustrated in **Figure 1**. The LSF and HSF images were matched for mean luminance (57 cd/m^2^) and RMS contrast in Matlab (The Mathworks, Natick, MA, United States), before they were fused to create hybrid face stimuli. Four sets of F (fearful) and N (neutral) hybrid stimuli were created for each of the seven identities: F_LSF_–F_HSF_, F_LSF_–N_HSF_, N_LSF_–F_HSF_, and N_LSF_–N_HSF_. Example hybrid stimuli are presented in **Figure 2**. A phase-scrambled neutral face (luminance and RMS contrast matched) was presented during the baseline period. The tasks were created and presented using VPixx software (version 3.15, VPixx Technologies, Montreal, QC, Canada), and displayed on a 27 cm × 48 cm LCD monitor with linearized output, at a viewing distance of 70 cm. Face images were centrally displayed in a 20 × 19.5 degree (500 × 700 pixels) mid-gray frame (47 cd/m^2^) on a gray background (65 cd/m^2^).

### Multifocal VEP

A 9-patch dartboard stimulus was programmed in VPixx, with a 4-degree central patch and two outer rings of four patches. Each patch fluctuated between two gray levels (70% Michelson contrast). The luminance for each patch was updated every video frame (60 Hz), following a pseudorandom binary m-sequence (*m* = 14). The m-sequences for each patch were maximally offset, so we could record independent responses. For the purpose of this paper, only the central patch was analyzed. The m-sequences were broken into four approximately 1-min recording segments. Participants were instructed to blink and rest in between recordings, and to maintain careful fixation during the recordings.

### Procedure

Prior to the task, participants were shown example hybrid stimuli. Although participants were not explicitly informed about the different hybrid face conditions, they were told that some faces might appear distorted, due to different spatial frequency content. During the experiment, a behavioral task was used to confirm participants were attending to the emotional content of the hybrid faces. Participants were verbally instructed that a hybrid face image would appear on screen for 500 ms, and after the image disappeared, they were to make 2AFC RESPONSEPixx button box decision between neutral and fearful expressions, where red button = *neutral* and green button = *fear*. Participants were instructed that this was not a timed response test, but that they should respond as accurately as possible, and guess when they were unsure of the facial expression. Participants were instructed to respond with their preferred hand, however, handedness was not measured. Following response selection, a phase-scrambled face was presented during a 1.8 s inter-stimulus interval.

To prevent fatigue, the recording was split into two blocks of 100 trials, so in total there were 50 trials for each of the four hybrid face conditions. Stimulus presentations were randomized, with set constraints on the number of trials for each hybrid condition. Trials with more than one button response were identified and removed from the analyses. For the second experiment, participants were instructed to passively view the multifocal dartboard stimulus. The m-sequences were broken into four approximately 1-min recording segments. Participants were instructed to blink and rest in between recordings, and to maintain careful fixation during the recordings.

### EEG Recordings and Analyses

Electroencephalogram was recorded from parietal and occipital sites (Oz, O1, O2, P3, P4, P5, P6, P7, P8, PO1, PO2, PO3, PO4, PO5, PO6, PO7, PO8) using a 32 channel Quickcap recording cap (Neuroscan, Compumedics). The data were band-pass filtered from 0.1 to 200 Hz, and sampled at 1 KHz. The ground electrode was positioned at AFz and linked mastoid electrodes served as a reference. Eye blinks were monitored with EOG electrodes, attached to the sub and supra orbital regions of the right eye.

Data analysis was performed with Brainstorm (Tadel et al., 2011), which is documented and freely available for download online under the GNU general public license^1^. Data were band-pass filtered (1–30 Hz) and signal space projection was applied to reduce eye-blink artifact. Segments of data containing low-frequency artifact were excluded from the analysis. For the facial emotion experiment, we extracted ERP epochs from -200 ms pre to 450 ms post-stimulus presentation. Baseline corrections were made for each epoch, by subtracting the mean amplitude present during the 200 ms period before stimulus presentation (i.e., -200 to 0 ms). Any epochs containing high amplitude noise (>75 μV) were excluded from the analysis. Separate ERP averages were computed for the low and high AQ groups, for each hybrid face condition: F_LSF_–F_HSF_ (Low AQ: 730 epochs, High AQ: 820 epochs)_,_ N_LSF_–N_HSF_ (Low AQ: 744 epochs, High AQ: 817 epochs), F_LSF_–N_HSF_ (Low AQ: 728 epochs, High AQ: 813 epochs) and N_LSF_–F_HSF_ (Low AQ: 744 epochs, High AQ: 819 epochs).

Consistent with previous research (Vlamings et al., 2009, 2010) visual inspection revealed P100 amplitudes were the greatest at electrodes P8, PO8, PO7, P7, O1, O2 and Oz, and N170 amplitudes were the greatest at P8, PO8, PO7, P7. To improve signal to noise ratio, the mean cluster responses were extracted. To reduce high frequency noise in these pre-processed waveforms, an additional 10 Hz low-pass filter was applied, prior to extracting the waveforms for the statistical amplitude and latency comparisons (Vlamings et al., 2010). The additional low-pass filter did not distort the P100 and N170 traces, indeed similar patterns of results were obtained regardless of whether this filter was applied. However, this step enabled more robust estimation of peak latencies at the individual level.

P100 and N170 amplitudes and latencies were detected using LabVIEW (National Instruments). P100 was detected as the maximum amplitude within the 90–150 ms time window, and N170 was detected as the minimum amplitude within the 160–240 ms time window. Peak amplitudes can be affected by noise, and emotion effects are not limited to the peaks, so we used the area under the P100 and N170 waveforms (60 ms window) as a measure of amplitude in the statistical comparisons (Vlamings et al., 2010). Due to individual differences in peak latencies, 60 ms time windows for P100 and N170 area measurements were centered separately on their peak latencies for each participant.

For the non-linear VEP analyses, custom Matlab/Brainstorm scripts were written to extract K1, K2.1 and K2.2 kernels for the central patch of the dartboard stimulus. The first order kernel (K1) is the difference in response when the patch was light (S1) or dark (S2) throughout the m-sequence, i.e., 0.5^∗^(S_1_ - S_2_). As described in previous papers (Klistorner et al., 1997; Jackson et al., 2013), the first slice of the second order kernel (K2.1) compares consecutive frames when a transition did and did not occur, i.e., K2.1 = 0.25^∗^(S_11_ + S_22_ - S_12_ - S_21_). The second slice of the second order kernel (K2.2) is similar, but compares frames with an interleaving frame of either polarity. In other words, K2.1 measures neural recovery over one frame (16.67 ms on a 60 Hz monitor) and K2.2 measures neural recovery over two frames (33.33 ms).

## Results

**FIGURE 3:**
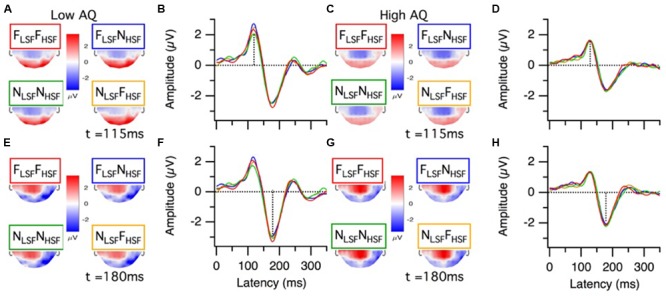
Grand average ERP results from the low and high AQ groups. P100 results for the low AQ group (*n* = 16) are presented as **(A)** topographies and **(B)** grand mean cluster waveforms (P8, PO8, PO7, P7, O1, O2, and Oz, cluster function = mean). Likewise, P100 results for the high AQ group (*n* = 17) are presented as **(C)** topographies and **(D)** grand mean cluster waveforms. N170 results for the low AQ group are presented as **(E)** topographies and **(F)** grand mean cluster waveforms (P8, PO8, PO7, P7, cluster function = mean). Likewise, N170 results for the high AQ group are presented as **(G)** topographies and **(H)** grand mean cluster waveforms. Results for the different hybrid face conditions are presented in different colors: F_LSF_–F_HSF_ (red), N_LSF_–N_HSF_ (green), F_LSF_–N_HSF_ (blue), and N_LSF_–F_HSF_ (orange). The top halves of the topography traces are occluded because we only recorded from a posterior set of electrodes (see Materials and Methods).

**FIGURE 4:**
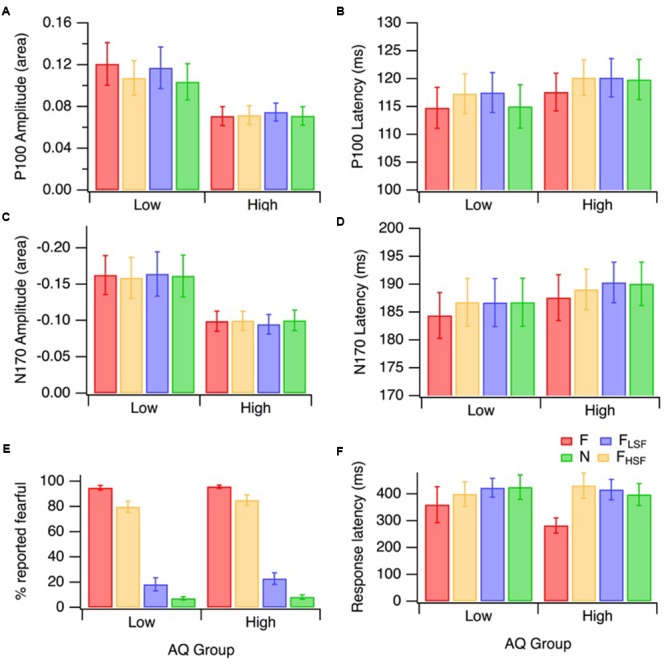
Mean ERP and behavioral responses for the low (*n* = 16) and high (*n* = 17) AQ groups to the different hybrid face sets: F_LSF_–F_HSF_ (red), N_LSF_–N_HSF_ (green), F_LSF_–N_HSF_ (blue), and N_LSF_–F_HSF_ (orange). The error bars on all panels represent ±1 SE. P100 amplitudes and latencies are displayed in **(A)** and **(B)**. N170 amplitudes and latencies are displayed in **(C)** and **(D)**. Behavioral results for the percentage of trials in which fear was identified, and the associated reactions times are presented in **(E)** and **(F)**, respectively.

Grand mean ERP topographies and waveforms for the low and high AQ groups are presented in **Figure 3**. The P100 topographies for the low and high AQ groups (**Figures 3A,C**, respectively) show there was a bilateral, occipital positivity at 115 ms latency for each of the hybrid face conditions. The N170 topographies for the low and high AQ groups (**Figures 3D,G**, respectively) show there was a right lateralized negativity, with strongest activations at electrode sites P8 and PO8, for each of the hybrid face conditions. Means and standard deviations for the low and high AQ groups are presented in **Figure 4**, with comparisons for P100 amplitude and latency, N170 amplitude and latency, and behavioral performance. As explained in the analyses section, we defined ERP amplitude as the area under the waveform (peak or trough latency ± 30 ms); however, it should be noted that we observed a similar pattern of results when we compared the waveform areas or the peak voltages across hybrid face conditions and AQ groups.

### Behavioral Performance

The emotion identification data (i.e., the percentage of trials in which fearful expression was reported) are presented in **Figure 4E**. A mixed factorial design 2 (AQ group) by 4 (hybrid face condition) ANOVA (with Greenhouse–Geisser corrections for unequal variance) was performed on emotion identification. There was a significant main effect of hybrid face condition on fear identification [*F*(2.23,64.55) = 435.79, *p* < 0.001, $\eta_{p}^{2}$ine-formula> = 0.94]. There was no significant interaction between the effects of hybrid face condition and AQ group on the fear identification [*F*(2.23,64.55) = 0.31, *p* = 0.758]; nor was there a main effect of AQ group [*F*(1,29) = 0.82, *p* = 0.372]. This shows that both AQ groups performed similarly. As one would expect, planned comparisons showed that F_LSF_–F_HSF_ hybrids were identified as ‘fearful’ significantly more often than N_LSF_–N_HSF_ [*F*(1,29) = 2423.73, *p* < 0.001, $\eta_{p}^{2}$ = 0.99], F_LSF_–N_HSF_ [*F*(1,29) = 486.39, *p* < 0.001, $\eta_{p}^{2}$ = 0.94], and N_LSF_–F_HSF_ hybrids [*F*(1,29) = 29.45, *p* < 0.001, $\eta_{p}^{2}$ = 0.50]. However, it was interesting that observers reported fearful expression substantially more often in the N_LSF_–F_HSF_ condition than in the F_LSF_–N_HSF_ condition [*F*(1,29) = 341.05, *p* < 0.001, $\eta_{p}^{2}$ = 0.92]. This indicates that HSF information plays a more important role in conscious identification of facial emotion than LSF information. Please note that response latencies (**Figure 4F**) were not similarly analyzed because interpretation of the data could be confused by the stipulation that participants should wait until the stimulus disappeared before responding.

### P100 Amplitude

A mixed factorial design 2 (AQ group) by 4 (hybrid face condition) ANOVA was performed on P100 amplitude (area), with means displayed in **Figure 4A**. The analysis revealed a significant difference in mean P100 amplitude across the four hybrid face conditions, [*F*(3,93) = 3.86, *p* = 0.012, $\eta_{p}^{2}$ = 0.11]. Across hybrid conditions, the P100 amplitude was lower in the high AQ group than in the low AQ group, and this difference was approaching significance [*F*(1,31) = 4.08, *p* = 0.052, $\eta_{p}^{2}$ = 0.17], as can be seen in **Figure 4A**. The interaction between AQ group and hybrid face condition was significant, [*F*(3,93) = 3.02, *p* = 0.034, $\eta_{p}^{2}$ = 0.09].

As the effect of the hybrid face conditions on P100 amplitude area was different for the two AQ groups, separate ANOVAs were performed for each group. For the low AQ group, there was a significant effect of the hybrid face conditions on the mean P100 amplitude, [*F*(3,45) = 3.83, *p* = 0.016, $\eta_{p}^{2}$ = 0.20]. Analytical contrasts revealed that, as predicted, mean P100 amplitude was significantly greater for F_LSF_–F_HSF_ hybrids compared to N_LSF_–N_HSF_ hybrids, [*F*(1,15) = 10.75, *p* = 0.005, $\eta_{p}^{2}$ = 0.42]. Interestingly, while P100 amplitude was significantly lower with N_LSF_–F_HSF_ than F_LSF_–F_HSF_ faces [*F*(1,15) = 5.36, *p* = 0.035, $\eta_{p}^{2}$ = 0.26], P100 amplitudes were not significantly different for F_LSF_–F_HSF_ and F_LSF_–N_HSF_ faces [*F*(1,15) = 0.43, *p* = 0.523]. This suggests that the effect of fearful emotion on P100 amplitude is mostly due to the LSF fearful input. Contrary to our hypothesis on hybrid face differences, the low AQ group produced no significant amplitude area mean difference between F_LSF-_N_HSF_ and N_LSF-_F_HSF_ [*F*(1,15) = 2.87, *p* = 0.111].

The ANOVA for the high AQ group revealed that, as hypothesized, there was no significant mean difference in P100 amplitude area across the four hybrid face conditions [*F*(3,48) = 0.85, *p* = 0.473]. This suggests an overall lack of fear affect or spatial frequency modulation of the early P100 ERP component in high autistic tendency.

### P100 Latency

A mixed factorial design 2 (AQ group) by 4 (hybrid face condition) ANOVA was performed on P100 latency, with the means and standard errors displayed in **Figure 4B**. Greenhouse–Geisser corrections were applied when Mauchly’s Test of Sphericity was violated. There was a significant difference in mean P100 latency across the four hybrid face conditions for the whole sample [*F*(2.32,71.86) = 6.05, *p* = 0.002, $\eta_{p}^{2}$ = 0.16]. Planned contrasts revealed a faster P100 latency for F_LSF_–F_HSF_ compared to N_LSF_–F_HSF_ [*F*(1,31) = 12.94, *p* = 0.002 $\eta_{p}^{2}$ = 0.29] and F_LSF_–N_HSF_ [*F*(1,31) = 18.52, *p* < 0.001, $\eta_{p}^{2}$ = 0.37] hybrids, respectively. The mean P100 latency for fearful hybrids F_LSF_–F_HSF_ was faster than for N_LSF_–N_HSF_ hybrids, however, this comparison did not reach significance [*F*(1,31) = 3.23, *p* = 0.082, $\eta_{p}^{2}$ = 0.09] The hybrid face by AQ group interaction was not significant [*F*(2.32,71.86) = 0.99, *p* = 0.388]. This indicates that facial emotion affected P100 latency in similar ways for the two groups. The between-groups ANOVA was also not significant [*F*(1,31) = 0.448, *p* = 0.508]. In summary, the effect of hybrid faces on P100 latency was similar for the low and high AQ group, with the whole sample producing significantly quicker latency to fear displayed at both LSF and HSF.

### N170 Amplitude

A mixed factorial design 2 (AQ group) by 4 (hybrid face condition) ANOVA (with Greenhouse–Geisser correction) was performed on N170 amplitude area, with means displayed in **Figure 4C**. There was no significant difference in mean N170 amplitude across the four hybrid face conditions [*F*(2.17,67.37) = 0.13, *p* = 0.898]. While there was no significant interaction [*F*(2.17,67.37) = 0.77, *p* = 0.479]; there was a significant effect of AQ group on mean N170 amplitude [*F*(1,31) = 4.23, *p* = 0.048, $\eta_{p}^{2}$ = 0.12]. As illustrated in **Figure 4C**, across conditions, mean N170 amplitudes tended to be lower in the high AQ group.

### N170 Latency

A mixed factorial design 2 (AQ group) by 4 (hybrid face condition) ANOVA (with Greenhouse–Geisser correction) was performed on N170 latency, with means displayed in **Figure 4D**. The analysis revealed a significant difference in mean N170 latency, across the four hybrid face conditions in the whole sample [*F*(2.33,72.29) = 6.28, *p* = 0.002, $\eta_{p}^{2}$ = 0.17]. Planned contrasts of the whole sample revealed a significantly faster N170 latency in response to fearful hybrids compared with N_LSF_–N_HSF_ [*F*(1,31) = 12.19, *p* = 0.001, $\eta_{p}^{2}$ = 0.28], N_LSF_–F_HSF_ [*F*(1,31) = 11.18, *p* = 0.002, $\eta_{p}^{2}$ = 0.27] and F_LSF_–N_HSF_ hybrids [*F*(1,31) = 15.89, *p* < 0.001, $\eta_{p}^{2}$ = 0.34]. No significant interaction was observed [*F*(2.33,72.29) = 0.36, *p* = 0.733, $\eta_{p}^{2}$ = 0.01]. The between-groups ANOVA was also not significant [*F*(1,31) = 0.303, *p* = 0.586]. In summary, the effects of facial emotion on N170 latency were similar for the low and high AQ groups, with more rapid latency responses to hybrids with both LSF and HSF fear.

### Multifocal VEP

**FIGURE 5:**
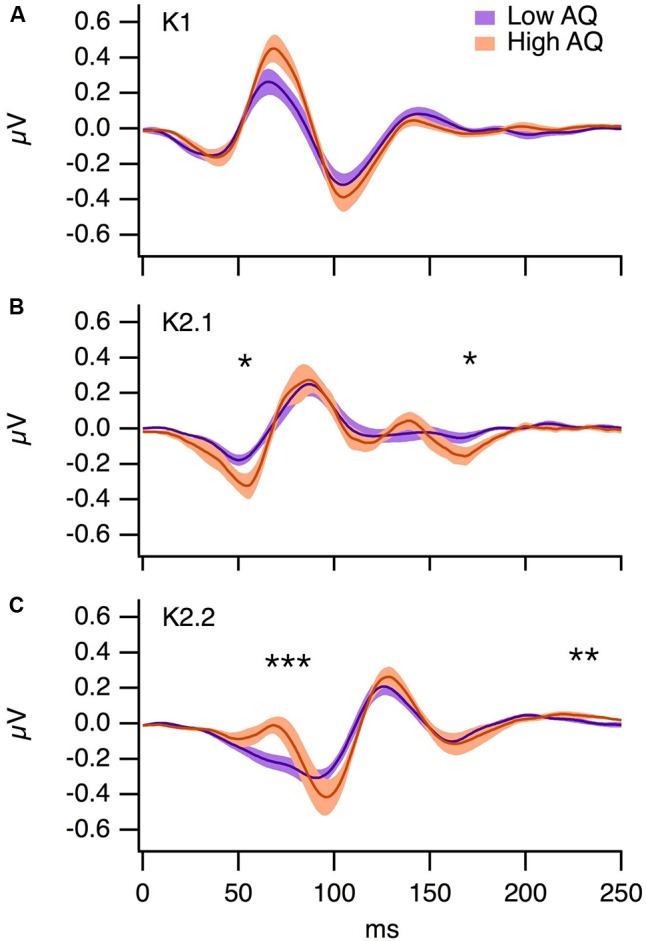
Grand averages from the low (*n* = 12) and high (*n* = 12) AQ groups for **(A)** K1, **(B)** K2.1, and **(C)** K2.2 VEP kernels. The shading represents ±1 SE, ^∗^*p* < 0.05, ^∗∗^*p* < 0.01, ^∗∗∗^*p* < 0.001.

The grand average K1, K2.1, and K2.2 Wiener kernel responses to the central multifocal patch for the low and high AQ groups are illustrated in **Figure 5**. For each participant, we selected the electrode with the highest amplitude response (Oz, O1, O2, or POz). The majority of participants showed maximal VEPs at Oz (Low AQ: Oz *n* = 9, POz *n* = 1 O1 *n* = 2, High AQ: Oz *n* = 7, POz *n* = 4, O2 *n* = 1). Independent samples *t*-tests were performed on kernel responses from the low and high AQ groups. For K1, the P70 peak was slightly higher in the high AQ group, but this difference was not statistically significant (*p* > 0.05). For K2.1, the amplitude of the N60 was significantly greater in the high AQ group [*t*(22) = 2.09, *p* < 0.05], a negativity at approximately 180 ms was also lower in the high AQ group [*t*(22) = 2.29, *p* < 0.05]. The early component of the K2.2 [which reflects magnocellular processing (Jackson et al., 2013)] was significantly higher in amplitude for the high AQ group [*t*(22) = 2.26, *p* < 0.05], yet there were no significant between-group differences in the parvocellularly driven N95 or P150 waveforms. These larger amplitude second-order non-linearities in the high AQ group are consistent with previous findings (Jackson et al., 2013), which suggests less efficient neural recovery within the magnocellular pathway for observers with high AQ.

## Discussion

Here, we conducted two investigations in groups with low and high autistic tendency recruited from within the neurotypical population. Firstly, we asked whether fearful expression affects the P100 and N170 ERP responses in the same way for low and high AQ groups. As expected, fearful expression modulated P100 amplitude in the low AQ group, but not in the high AQ group. In the low AQ group, P100 amplitudes were significantly greater for F_LSF_–F_HSF_ faces than for N_LSF_–N_HSF_ faces. Interestingly, while P100 amplitude was significantly greater with F_LSF_–F_HSF_ than N_LSF_–F_HSF_ faces_,_ it was not significantly different with F_LSF_–F_HSF_ and F_LSF_–N_HSF_ faces. This suggests that the effect of fearful expression on producing greater P100 amplitude is mostly via fearful expression being carried by LSF. By contrast, in the high AQ group there was an overall reduction in P100 and N170 amplitude and there was no effect of fearful expression on ERP amplitudes. P100 and N170 latencies were faster for the F_LSF_–F_HSF_ hybrids than for the other conditions, but there were no significant latency differences between the two groups. We used Wiener kernel analysis of the visual evoked potential to investigate whether differences between the low and high AQ groups could reflect differences in magnocellular processing. The K2.1 non-linear VEP response amplitude was higher in the high versus low AQ group, which suggests inefficient neural recovery within the magnocellular stream (Sutherland and Crewther, 2010; Jackson et al., 2013).

We demonstrated that for people with low levels of autistic personality traits, fearful emotion tends to affect the P100 amplitude but not the N170 amplitude. Differences in the effects of our hybrid emotional stimuli on the P100 and N170 can be interpreted in terms of what we know about these early potentials. The literature suggests that P100 amplitude modulation reflects both rapid orienting to salient information (Hillyard and Anllo-Vento, 1998) and rapid identification of threat-related input, such as fearful facial expression (Batty and Taylor, 2003; Pourtois et al., 2004; Magnée et al., 2008; Feng et al., 2009; Luo et al., 2010; Forscher and Li, 2012; Meaux et al., 2013; Zhang et al., 2013). Consistent with this literature, our findings suggest that differences in facial emotion can be processed as early as the P100, but only in groups with low levels of autistic tendency. The N170 amplitude is sensitive to configural processing of faces, yet there are mixed findings as to whether it is influenced by facial emotion (Holmes et al., 2005; Pourtois et al., 2005). Vlamings et al. (2009) found increased N170 amplitude responses to fearful hybrids compared to neutral hybrids, yet in our experiment, these differences were not observed for either AQ group. On the contrary, we did observe some latency differences for the whole sample, with faster N170 responses for F_LSF_–F_HSF_ hybrids than for the other hybrid conditions. The slower N170 responses for the hybrids with mixed expressions (i.e., F_LSF_–N_HSF_ and N_LSF_–F_HSF_) might reflect disruptions in configural processing of these stimuli (Bentin et al., 1996).

Consistent with Stavropoulos et al. (2016), the P100 and N170 amplitudes were lower in the high AQ group than in the low AQ group, yet contrary to their findings, we did not observe any clear P100 or N170 latency differences between the two AQ groups. There are several differences between our experiments that may explain these differences in results. First of all, we used hybrid face stimuli, whereas they used unaltered (i.e., broadband spatial frequency) face stimuli. Secondly, they observed the greatest between-groups differences when the stimuli were presented non-consciously (16 ms presentation time), whereas our stimuli were presented for 500 ms. Finally, their high AQ sample had very low amplitude/high noise ERP results. This may imply that their high AQ sample had greater face processing impairments than our high AQ sample. Given that we recorded clear P100 and N170 responses from both groups, we were able to make meaningful comparisons for the effects of facial emotion on ERP responses in the low and high AQ neurotypical groups.

Differences in the effects of facial emotion on P100 responses for the low and high AQ groups may reflect underlying differences in magnocellular function. In the low AQ group, P100 amplitudes were greater for F_LSF_–F_HSF_ than N_LSF_–F_HSF_ faces_,_ yet there was no difference in P100 amplitude for F_LSF_–F_HSF_ and F_LSF_–N_HSF_ faces. This is consistent with evidence from Vlamings et al. (2009) that LSF input contributes to rapid detection of fearful expression. Recent studies have demonstrated magnocellular projections from the pulvinar to the orbitofrontal cortex, which allow for rapid feedback to bias visual processing toward behaviorally relevant stimuli (Bar et al., 2006; Kveraga et al., 2007). Given the spatial frequency preferences of the magnocellular and parvocellular pathways (Livingstone and Hubel, 1988), our results are consistent with evidence that the effects of fearful expression on early visual processing are likely to be carried by the magnocellular pathway. The K2.1 non-linear VEP response amplitude was higher in the high AQ group, which suggests inefficient neural recovery within the magnocellular stream (Sutherland and Crewther, 2010; Jackson et al., 2013).

In addition, fearful expression did not tend to affect P100 amplitudes in the high AQ group in any hybrid condition, compared to the low AQ group, which demonstrated sensitivity to hybrid conditions. This suggests that even within the neurotypical population, very early processing of fear-related input varies for individuals with different levels of autistic personality traits. While the exact nature of magnocellular impairment in high autistic tendency individuals remains unresolved (Simmons et al., 2009; Thye et al., 2017); the differences in early ERP responses to fearful faces that we observed for low and high AQ groups could reflect a magnocellular difference in processing LSF facial emotion.

Our results for the non-linear VEP analysis are consistent with magnocellular differences between the low and high AQ groups. Previous investigations of the contrast-response functions for non-linear VEP components (Klistorner et al., 1997; Jackson et al., 2013) indicate that the K2.1 and early components of the K2.2 waveform are of magnocellular origin, whereas the later K2.2 components are of parvocellular origin. Our results are consistent with previous findings that magnocellularly driven VEP non-linearities tend to be greater in groups with high AQ, but parvocellularly driven VEP non-linearities tend to be similar for both groups (Jackson et al., 2013). This implies that the magnocellular pathway recovers less efficiently from rapid stimulation in individuals with high AQ than in individuals with low AQ. Our results suggest that magnocellular projections, which would normally enable rapid detection of threatening stimuli, are less efficient within the broader autistic phenotype. This may contribute to explaining why the high AQ group produced reduced P100 ERP responses to all hybrid face conditions.

The results of our behavioral task are seemingly at odds with our ERP results. Both high and low AQ groups performed similarly in detecting fearful expression in the F_LSF_–F_HSF_, F_LSF_–N_HSF_, N_LSF_–F_HSF,_ and N_LSF_–N_HSF_ hybrid faces. More interestingly, our results indicated that both AQ groups relied upon HSF information to recognize fearful expressions. This HSF preference, however, was elicited in a task that required a 500 ms wait time before response. The conscious detection and report of fearful expression within a hybrid face, as described in prior studies of conscious perception, has demonstrated reliance on HSF facial information (Williams et al., 2004). However, these behavioral measures are not sensitive to very early visual processing stages that precede conscious awareness. We demonstrated differences in ERP responses to fearful and neutral face stimuli as early as 100 ms post-presentation in the low AQ group, but not in the high AQ group. Our non-linear VEP experiment found no significant between-group differences in the parvocellularly driven K2.2 N95 or P150 waveforms, which suggests the differences in the fearful face P100 are magnocellularly driven.

## Conclusion

In conclusion, for observers with low AQ, we found that fearful expression enhances rapid electrophysiological responses to faces. Our results suggest that this enhancement is likely to occur via rapidly processed, magnocellular input. For observers with high AQ, we did not find any difference in ERP responses to fearful and neutral faces. Consistent with previous studies (Jackson et al., 2013), our high AQ group showed abnormal temporal processing in the magnocellular pathway. These results support the notion that autism involves differences in processing of LSF information. Our results suggest that magnocellular projections, which would normally enable rapid detection of threatening stimuli, are not utilized efficiently for those with higher autistic tendency. Hence, visual processing differences may underlie some of the socio-cognitive aspects of autism.

## Author Contributions

AB contributed to this manuscript, including creating the first experimental design, conducting literature review, participant testing sessions, EEG and behavioral analysis and the preparation of this manuscript. LH contributed this manuscript by creating the second experimental design, programming analysis, EEG analysis of second experiment, creating manuscript figures, manuscript section for second experiment and manuscript editing. TF-B contributed by assisting in research participant testing sessions. DC contributed by supervising second experiment design and manuscript editing.

## Conflict of Interest Statement

The authors declare that the research was conducted in the absence of any commercial or financial relationships that could be construed as a potential conflict of interest.

## Footnotes

1 http://neuroimage.usc.edu/brainstorm
